# Knowledge Transfer and Networking Upon Implementation of a Transdisciplinary Digital Health Curriculum in a Unique Digital Health Training Culture: Prospective Analysis

**DOI:** 10.2196/51389

**Published:** 2024-04-15

**Authors:** Juliane Kröplin, Leonie Maier, Jan-Hendrik Lenz, Bernd Romeike

**Affiliations:** 1 Department of Oral and Maxillofacial Surgery, University Medical Centre Rostock, Rostock, Germany; 2 Department of the Dean of Studies in Medical Didactics, University of Rostock, Rostock, Germany

**Keywords:** big data, digital didactics, digital health applications, digital leadership, digital literacy, generative artificial intelligence, mobile working, robotics, telemedicine, wearables

## Abstract

**Background:**

Digital health has been taught at medical faculties for a few years. However, in general, the teaching of digital competencies in medical education and training is still underrepresented.

**Objective:**

This study aims to analyze the objective acquisition of digital competencies through the implementation of a transdisciplinary digital health curriculum as a compulsory elective subject at a German university. The main subject areas of digital leadership and management, digital learning and didactics, digital communication, robotics, and generative artificial intelligence were developed and taught in a transdisciplinary manner over a period of 1 semester.

**Methods:**

The participants evaluated the relevant content of the curriculum regarding the competencies already taught in advance during the study, using a Likert scale. The participants’ increase in digital competencies were examined with a pre-post test consisting of 12 questions. Statistical analysis was performed using an unpaired 2-tailed Student *t* test. A *P* value of <.05 was considered statistically significant. Furthermore, an analysis of the acceptance of the transdisciplinary approach as well as the application of an alternative examination method (term paper instead of a test with closed and open questions) was carried out.

**Results:**

In the first year after the introduction of the compulsory elective subject, students of human medicine (n=15), dentistry (n=3), and medical biotechnology (n=2) participated in the curriculum. In total, 13 participants were women (7 men), and 61.1% (n=11) of the participants in human medicine and dentistry were in the preclinical study stage (clinical: n=7, 38.9%). All the aforementioned learning objectives were largely absent in all study sections (preclinical: mean 4.2; clinical: mean 4.4; *P*=.02). The pre-post test comparison revealed a significant increase of 106% in knowledge (*P*<.001) among the participants.

**Conclusions:**

The transdisciplinary teaching of a digital health curriculum, including digital teaching methods, considers perspectives and skills from different disciplines. Our new curriculum facilitates an objective increase in knowledge regarding the complex challenges of the digital transformation of our health care system. Of the 16 student term papers arising from the course, robotics and artificial intelligence attracted the most interest, accounting for 9 of the submissions.

## Introduction

### Background

With the Digital Healthcare Act (German: Digitale-Versorgung-Gesetz), the spectrum of digitalization in the health care system was expanded in Germany in 2019. It includes, among others, the promotion of telemedicine and the expansion of the telematics infrastructure. In addition, a legal framework was created, which, for the first time, entitles insured persons to digital health applications. Digital health applications belong to low-risk medical devices and are primarily intended to support the detection, monitoring, treatment, or alleviation of diseases, injuries, or disabilities. Since January 2021, patients have also been entitled to have access to their data, which have generated during hospital treatment and stored in their electronic patient record. This facilitates electronic provision of medical information, in particular findings, diagnoses, treatment measures carried out and planned, as well as treatment reports for use across facilities, disciplines, and sectors [1,2].

These and further developments show that digital health is creating a new form of health care and is changing the way medicine is delivered and managed [3].

For medical educators, this evolution presents a 2-fold challenge: first, to understand and keep up with the rapidly evolving digital health landscape; and second, to effectively integrate this knowledge into medical curricula to prepare the next generation of health care professionals. Recognizing this gap and the opportunity it presents, the implementation of a comprehensive digital health curriculum is paramount.

Previous studies have suggested that digital health education should be integrated into medical school curricula, with a special emphasis on topics related to knowledge, skills, and attitudes [4].

Several other studies have emphasised the need for medical schools to prepare students for a future in digital health by incorporating digital health competencies into their curricula [4-7].

However, the transdisciplinary approach within university (digital) teaching is still not widespread. The need for such an approach arises from the potential for innovation [8] and is based on professional policy framework conditions such as the new dental licencing regulations [9]. Elective classes seem to be suitable formats for timely introduction, but a longitudinal implementation in mandatory curricula should be the goal [5].

### The Implementation of a Transdisciplinary “Digital Health” Curriculum at Our University

The curriculum “Digital Health - Digitalisation and Digital Transformation of Medicine” was offered for the first time at our university in the winter semester of 2022-2023. Students from all faculties and all semesters of the university were eligible to participate.

The learning objectives were developed on the basis of existing literature [4-6,10] and interviews with transdisciplinary experts in the areas of human medicine, dental medicine, medical didactics, computer science, business administration, theology, and ethics. The curriculum is divided into the 4 subareas of digital didactics, namely digital communication, management and digital leadership, and robotics and generative artificial intelligence (AI), each with 14 weekly lessons as well as an introductory event and a final examination and evaluation event. The lessons particularly encompassed the following topics: augmented or virtual reality, big data or generative AI, data protection or information security, digital leadership, digital didactics, ethical aspects of digital health, new work, robotics, social media, open educational resources, digital health applications, wearables, simulation training, and telemedicine (Table 1).

**Table 1.** Digital health curriculum.

Topics	Goals, subareas, and time
Digital communication	**Goal:** knowledge transfer regarding modern communication systems, consideration of legal framework conditions, and ethical aspects during transdisciplinary implementation and application **Subareas:** telemedicine, digital patient files, ethics, messenger apps, digital health applications **Time:** 3 lessons, each lasting 90 minutes
Digital didactics	**Goal**: application of modern teaching and learning methods and creating a nondiscriminatory framework for studies **Subareas**: open educational resources, virtual or augmented reality, simulation training **Time**: 3 lessons, each lasting 90 minutes
Management and digital leadership	**Goal:** knowledge transfer regarding digital transformation including economic aspects as well as the importance of innovative leadership styles **Subareas:** leadership, information security, data protection, economy, social media, and mobile working **Time:** 3 lessons, each lasting 90 minutes
Robotics and artificial intelligence	**Goal:** knowledge transfer about possible applications of surgical robots, individualized medicine, and possible uses of generative artificial intelligence in teaching, research, and patient treatment **Subareas:** robotics, generative artificial intelligence, wearables, and big data **Time**: 3 lessons, each lasting 90 minutes

The aims of this digital health curriculum are as follows: (1) integrating basic digital health content into the curriculum of a university in northern Germany; in a transdisciplinary approach, students will be taught the necessary competencies to be able to apply digital health technologies in their later work; (2) considering the new licencing regulations for dentists; dental students, in particular, should be encouraged to use the newly implemented compulsory elective subject to gain knowledge in the field of digital health; and (3) to encourage students to critically engage with the topic of digital health within the framework of a scientific thesis; this also intended to reflect currently relevant digital health topics from the students’ perspective as a basis for further curriculum development.

The curriculum contents were taught over a period of 1 semester within the framework of a compulsory elective subject.

Furthermore, this study aims to analyze the objective acquisition of digital competencies through the implementation of a transdisciplinary digital health curriculum at a German university.

## Methods

### Ethical Considerations

The study has been reviewed by the ethics committee of the Faculty of Medicine of the University of Rostock, Germany, and has been approved (A 2022-0137).

### Demographics and Previous Teaching of Digital Health

Student-related data about educational level, gender distribution, and career goals were analyzed. At the beginning of the semester, students were asked whether digital health learning objectives had already been taught in previous courses, using a Likert scale (1=very well taught to 5=not taught at all).

### Students’ Assessment and a New Examination Approach for Further Development

To measure the allocation of knowledge of the participants, the participants’ prior knowledge was assessed during the introductory lesson through a theoretical test (pretest) consisting of 12 questions. Ten questions were multiple-choice and 2 were open questions. The test was specifically related to the topics covered in the curriculum. Multiple-choice questions assessed knowledge on the topics of digital transformation, ethics, change management, data protection, robot-assisted surgery, digital patient files, video consultation, and simulation training. The didactics section was covered by 2 open questions and 1 multiple-choice question. At the final seminar, the theoretical test was repeated with similar questions (posttest).

In addition to the standardized questions, students were asked to write a scientific paper. The topic could be chosen independently. However, a prerequisite was a content-related reference to the overarching topic of digital health. The objectives of the examination are to (1) encourage students to critically engage with a digital health topic of their choice, (2) promote scientific work, and (3) obtain an insight into the topics of digital health perceived as relevant by the students as a basis for further curriculum development.

For further structuring of the curriculum, the scientific papers were assigned to one of the main topic areas based on the selected headings and abstract contents.

### Statistical Analysis

The data were analyzed using SPSS (version 27; IBM Corp) software. The gender distribution, career goals, intended subject area, and scientific papers were analyzed descriptively. Statistical analysis of pre-post test results and previous teaching of learning objectives was performed using an unpaired (learning objectives) and paired (pre-post test results) 2-tailed Student *t* test. A *P* value of <.05 was considered statistically significant.

## Results

### Educational Level of the Participants

Within the first year, a total of 20 students (5 in the winter term and 15 in the summer term) participated in the digital health curriculum. The average age of the participants was 22.3 (range 19-30) years. At the time of participation, 15 participants studied human medicine, 3 participants were studying dentistry, and 2 participants were studying medical biotechnology. In total, 11 (61.1%) students in human and dental medicine were in the preclinical phase and 7 (38.9%) were in the clinical phase.

### Gender Distribution

**Figure 1.**
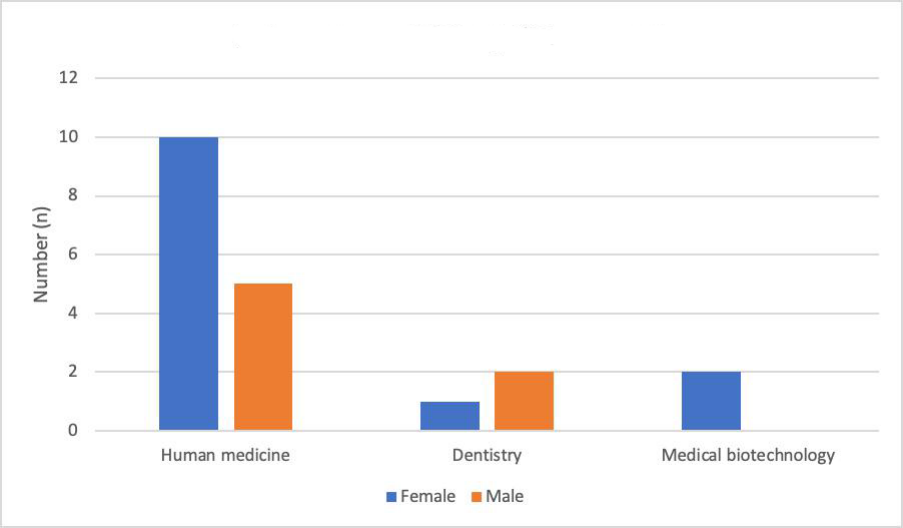
Gender distribution by study subject.

Figure 1 shows the gender ratio according to the subjects of study among the participants. In total, 13 participants were female and 7 were male. Among human medicine students, 10 were female and 5 were male. Two dentistry students were male and 1 was female. Both biotechnology students were female.

### Career Goals

Two questions were aligned with the focus on future professional activities. The first question asked whether the respondents wanted to work in an inpatient or outpatient setting. The options “other” and “don’t know yet” could also be selected. Furthermore, the students were asked about their desired goal of becoming a specialist doctor. As shown in Multimedia Appendix 1, the majority of participants are still undecided on whether they want to work in the outpatient or inpatient sector in future. Multimedia Appendix 2 shows the answers to the question about the goal of becoming a medical specialist, which was answered by the participating human medicine students. According to this, most of the participants who already know their career goal intended to become a specialist in surgery (n=4).

### Previous Teaching of Digital Health

During the first lesson, students were asked whether digital health learning objectives have already been taught in previous courses, using a Likert scale (1=very well taught to 5=not taught at all). Table 2 shows the corresponding evaluations.

**Table 2.** Evaluation of the learning objectives of previous teaching of the digital health curriculum.

Learning objectives	Clinical, mean	Preclinical, mean	*P* value
All	4.2	4.4	.02
Big data	4.9	4.7	.36
Artificial intelligence	4.7	4.6	.84
Digital health applications	4.1	4.3	.80
Messenger apps	4.3	4.7	.26
Wearables	4.4	4.8	.18
Telemedicine	4.1	4.7	.20
Data protection and information security	3.6	3.7	.82
Digital ethics	4.0	4.1	.87
Simulation training	3.9	4.5	.28
Virtual or augmented reality	3.8	4.6	.10
Economy	3.7	4.6	.09
Digital didactics	3.0	3.6	.41
Robotics	4.6	4.5	.83
Digital leadership	4.6	4.8	.46
Mobile working	4.7	4.7	.97
Social media	4.6	4.4	.69
Open educational resources	3.9	4.3	.55

Among clinical students, significantly better overall coverage of the digital health learning objectives is evident.

### Pre-Post Test Results and Term Paper Evaluation

In the pretest, the participants scored an average of 4 points compared to 8.3 points in the posttest. Consequently, there was a significant increase of 106% in knowledge (*P*<.001; Table 3).

**Table 3.** Increase in knowledge determined via a pre-post test (maximum achievable score 12; 106% increase in knowledge by 4.3 points; *P*<.001).

Increase in knowledge	Pretest^a^	Posttest^b^
Total score	4.0	8.3
Clinical	4.1	8.7
Preclinical	4.2	8.2
Female participants	3.6	8.3
Male participants	4.7	8.1

a Difference in pretest scores between clinical and preclinical participants: *P*=.96; differences in posttest scores between clinical and preclinical participants: *P*=.38.

b Difference in pretest scores between male and female participants: *P*=.11; difference in posttest scores between male and female participants: *P*=.17.

Neither gender nor study phase affected pre- or posttest results. As shown in Table 4, the most frequently selected main topic was robotics and AI.

**Table 4.** Digital health topics selected by students for their term papers.

Titles of the students’ term papers	Digital health main topic
Progress of computer-assisted procedures and robotics in implantology	Robotics and AI^a^
Mind reading with functional magnetic resonance imaging and AI	Robotics and AI
To what extent can the Da Vinci Robot help reduce postoperative complications?	Robotics and AI
Algorithms against prejudice? The role of AI in combating gender discrimination in the health sector	Robotics and AI
What opportunities arise from the use of AI in medicine and what are the associated problems?	Robotics and AI
Applications of AI in Radiology	Robotics and AI
AI and robotics in Orthopaedics and Trauma Surgery	Robotics and AI
Opportunities and limits of AI in the health sector	Robotics and AI
Use of AI for early detection of dementia	Robotics and AI
Data ethics in the digital world	Management and leadership
Does digitalisation in medicine lead to a loss of skills and knowledge among medical staff?	Digital didactics
“Flipped Classroom”: Possibilities of redesigning of an accompanying seminar on the study of human medicine.	Digital didactics
Implementation of an interdisciplinary elective subject “Digital Health”	Digital didactics
Aspects of discrimination against older people in digital medicine	Digital communication
What role do chatbots play in medical studies	Digital communication

a AI: artificial intelligence.

## Discussion

### Overview

Current social, political, and economic developments in Germany require a reorientation of university teaching, considering digital learning and teaching strategies. The necessity is also reflected in the restructuring of established framework conditions, such as the amendment of dental and medical licencing regulations [9,11].

This study aimed to analyze the objective acquisition of digital competencies through the implementation of a transdisciplinary digital health curriculum at a German university.

The learning objectives were imparted on the main topics of management and digital leadership, robotics and AI, digital communication, and digital didactics within the framework of a 1-semester curriculum. Objective knowledge gain was determined using a pre-post test design. In addition, the extent to which the approach of transdisciplinary networking could be implemented was analyzed. This was quantified by the disciplines and the number of clinical and preclinical participants. Overall, the results were analysed over 1 year (2 cohorts). In the second run, the number of participants has already tripled.

### Characterization of the Participants

According to the Federal Statistical Office, 64.8% of students in human medicine in 2021 were female [12]. This corresponds to the distribution of participants in our curriculum, even when considering the isolated subject group of human medicine being the most frequently represented. Consequently, it can be assumed that the topic is not gender-specific and is of equal interest to male and female students. This cannot be confirmed for participants from the fields of dentistry and medical biotechnology. However, the small number of participants must be considered here.

### Previous Teaching of Digital Health

Evaluation of the students at the beginning of the semester revealed that all the content of the curriculum has not been taught at all or only to a very limited extent. Even though there was a significant difference in knowledge between the clinical and preclinical sections, this concerns all participants. Consequently, it can be assumed that this deficit will not be sufficiently compensated for in higher semesters with regard to the clinical phase.

The results also indicate that most participants are still open about their career goals. This applies both to the future field of work (outpatient vs inpatient) and to the intended specialization. Therefore, the general approach to teaching content can be considered suitable.

### Assessment of the Increase in Knowledge

As reported by studies with a similar study design, a significant objective increase in knowledge could be achieved among participants through the curricular dissemination of knowledge on relevant digital health topics. It should be noted that some students participated in the curriculum out of interest in the content but without aiming to achieve a good grade. Consequently, it can be assumed that some students did not prepare for the posttest. The fact that summative assessment of the intended learning objectives at the beginning of the curriculum increases learning success has previously been described [13].

Regarding the current evidence in the development of digital literacy, the focus is increasingly on social interaction and lifelong learning skills in an innovative teaching and learning culture, in addition to subject knowledge [5].

### Term Paper Evaluation

When analyzing the selected term papers, it quickly became clear that the topic of AI is of outstanding importance among digital health topics. This seems to be explained, in particular, by the strong media presence of the topic. The rapid development of generative AI has received special media attention with the launch of ChatGPT in 2022 [14-16]. Two challenges arise, in particular, for the curriculum. Although the special importance of flexible and adaptive teaching formats to be able to integrate innovations into teaching without delay is becoming apparent, the establishment of framework conditions for the application of generative AI in teaching, research, and clinical practice is coming into focus. Both focal points and associated challenges were already considered and will be further developed for our future digital health curriculum.

### The Role of Leadership in a Digital Health Training Culture

Digital transformation is a continuous process that is better accepted by those who perceive digitalization as relevant to their own work. Digital leadership describes the special role of managers in the implementation of digital transformation. It is up to managers in the health care sector to align the strategic orientation to digital transformation with the company’s goals and needs and to create an appropriate digital culture. Regarding the provision of early access to the necessary knowledge on topics related to digital health, managers in the field of education have a special responsibility [17].

The transdisciplinary approach of the digital health curriculum acknowledges the current evidence for the success of digital transformation. In particular, evidence from economic evaluations has shown that in a networked environment, the opening of boundaries is necessary to create innovation and exploit synergies [8].

With an average value of 5 on the Likert scale, the results of the initial evaluation show that this knowledge has not yet been imparted in the participants’ previous curricula. Consideration of the transdisciplinary digital health curriculum is, therefore, of particular importance.

### Digitalization Connects: the Necessity of a Transdisciplinary Digital Health Curriculum

The goal of opening of the curriculum to all faculties is to expand the transdisciplinary network to promote an innovation-driven teaching and learning environment. This basic idea represents a unique selling point for previously established digital health curricula.

Our results indicate that this opportunity was already realized in the first year by students from 3 different disciplines, such as human medicine, dentistry, and medical biotechnology. The distribution of clinical and preclinical students also shows cross-semester interest.

In the future, an increase in the participation of dental medicine students is expected. This is due to the new orientation of the dental licencing regulations, which mandate participating in curricula by choosing from among the elective subject areas (to which the compulsory digital health elective subject is assigned), both for the preclinical and clinical study phases [9].

It should be noted, however, that only 5 out of 20 students did not belong to the field of human medicine. These results suggest that the transdisciplinary approach needs to be further promoted, addressed, and implemented to achieve an even better transdisciplinary exchange.

Social media use may present an opportunity for increasing the visibility of our transdisciplinary curriculum and its learning objectives. The curriculum is currently already accompanied by a social media channel. The importance of social media in teaching and research is currently the focus of social debates and scientific studies [18,19]. For better assessment of the importance of social media in a modern academic teaching and learning culture, the authors believe that further studies are needed.

### Emerging Technologies in a Transforming Health Care System

The use of modern technologies has enormous potential for optimizing patient treatment [20,21]. In surgery, in particular, there is a wide range of applications in the operating theater and perioperative management.

A recent editorial describes current emerging innovations with particular potential, which are also included in the digital health curriculum [20]. In particular, this involves the contents of machine learning–enabled clinical decision-making support, computer vision and augmented reality, as well as wearable devices and remote patient monitoring. The dynamic nature of these developments, among others, shows the particular importance of a flexible and adaptive curriculum to be able to integrate emerging technologies into teaching without delay.

Robot-assisted surgery, including approaches to telesurgery, is of particular importance, especially in surgery. The special importance of robotics for patient care has already been described several times and is now an integral part of numerous hospitals [22,23].

The special importance of robotics is also reflected in the selection of homework topics. Three of the 16 papers submitted focus on robotics in medicine.

However, the increasing use of robotics in the operating theater also requires special skills that can and must be practised extensively in a simulation-based setting [24]. This requires time and financial resources, as well as training in a supervised setting [25]. In teaching and further education, these prerequisites represent a hurdle. In particular, cost-intensive virtual and augmented reality simulators are often only rarely available; their use in teaching is generally yet not structured [26]. User acceptance is indisputably high and can increase satisfaction in addition to learning success [27]. However, the topic requires economic reflection and a basic understanding of project management—an aspect that was addressed in the curriculum section of Management and Digital Leadership.

In addition to the implementation and continuous further development of technical innovations in clinical applications, achievements with disruptive innovation power also play a special role in future teaching and research. The disruptive potential of digital transformation is currently manifesting itself in particular in the launch of generative AI, such as ChatGPT [14].

### Generative AI, Web-Based Meetings, and the Challenge of Flexible Adaptive Training

The examination of digital teaching methods has experienced a surge in innovation, particularly in the context of the COVID-19 pandemic [28]. Experience in the field of telemedicine has provided a blueprint for web-based teaching with simultaneous integration of knowledge content in telemedicine. Thus, knowledge transfer could be extended by the achievement of local flexibility [28].

But approaches that account for time flexibility are also described: the “flipped classroom” model, for example, is an approach to active self-directed learning in which students acquire the basic concepts themselves before class—for example, through recorded lectures or interactional learning modules provided by a learning management system—so that class time can be used for active learning activities such as exercises, projects, or discussions. Valuable time spent in presence is used for the application, rather than acquisition, of knowledge. This can increase both student performance and student satisfaction [29,30].

In addition to the flexibility of location and time, there are often limits to accessing real-world working environments. To be able to train practical and theoretical skills in a realistic setting, such as an operating theater, teaching using virtual and augmented reality offers promising potential.

Virtual reality refers to complete visual immersion in an artificial, computer-generated environment. In augmented reality, holograms, which often also enable interaction, appear projected into the room through semitransparent glasses. Mixed reality is the combination of digital screens with projected interactional holograms. The user sees the real world while simultaneously manipulating the digital content generated by the device [31].

Both technologies are increasingly being integrated in the clinical setting, but also in teaching, such as the visualization of organs. In clinical applications, augmented reality enables the simulation of patient encounters to train communication skills or intraoperative decision-making to increase safety during surgery [32].

### Limitations

This study’s limitations particularly include its single-center design and the small number of participants at the time of analysis. In addition, the final test only examined excerpts from topics that cannot represent the full scope of the curriculum. The choice of term paper is also subject to numerous influencing factors, so the motivation for choosing the topic cannot be clearly identified.

### Conclusions

This study aims to analyze the objective acquisition of digital competencies through the implementation of a transdisciplinary digital health curriculum at a German university. The results show that relevant content on digital health topics has not been taught sufficiently at the university outside our new digital health curriculum. The objective increase in the knowledge on these topics within the framework of the digital health curriculum could be verified as significant via a pre-post test design.

The approach of transdisciplinary development of a digital health curriculum seems especially promising. We provided dentistry students a platform to complete their recently compulsory elective subject. We observed that dentistry students could complete their recently compulsory elective subject when using an appropriate digital platform.

The integration of written assignments as a special examination element can promote critical engagement with digital health content. This also facilitates gaining insight into digital health topics and issues that are relevant to students. We can harness these insights in further developing our curriculum.

Together with the current literature, our data indicate that the content of digital health curricula must be transferred into standard teaching for all health science students.

## Supplementary material

10.2196/51389 Multimedia Appendix 1 Career goals.

10.2196/51389 Multimedia Appendix 2 Aspired specialist of the human medicine participants.

## Abbreviations

AI: artificial intelligence
