# Supplementary figures and images for: Knowledge Transfer and Networking Upon Implementation of a Transdisciplinary Digital Health Curriculum in a Unique Digital Health Training Culture: Prospective Analysis
Source: JMIR Med Educ. 2024 Apr 15;10:e51389. doi: 10.2196/51389 (PMC11034421; doi:10.2196/51389)

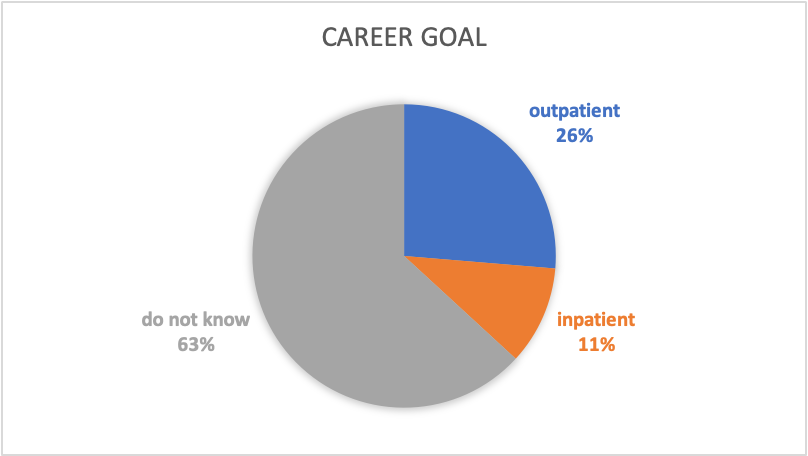

Supplement: Multimedia Appendix 1 [file mededu-v10-e51389-s001.png]

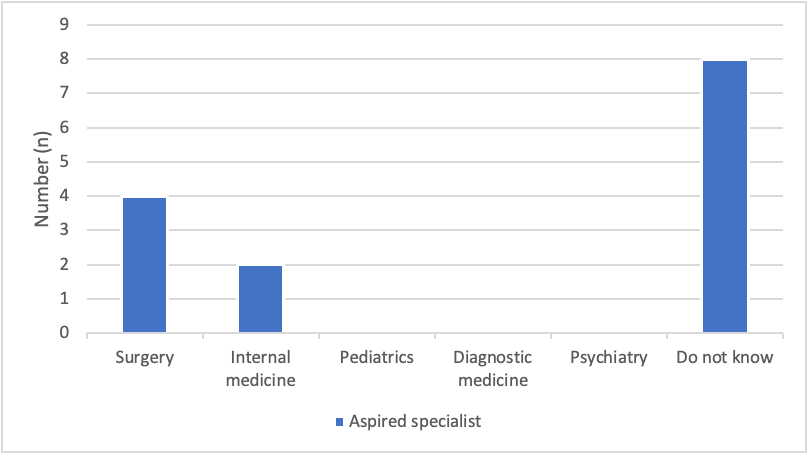

Supplement: Multimedia Appendix 2 [file mededu-v10-e51389-s002.png]
